# Human costal cartilage, tooth cavities, and femur nutrient canals—new niches for insects used in forensic entomology

**DOI:** 10.1093/fsr/owae028

**Published:** 2024-04-23

**Authors:** Marcin Tomsia, Andrzej Grzywacz, Krzysztof Szpila, Kinga Walczak, Karolina Mahlerová, Daniel Vaněk, Szymon Matuszewski

**Affiliations:** Department of Forensic Medicine and Forensic Toxicology, Faculty of Medical Sciences (FOMS) in Katowice, Medical University of Silesia in Katowice, Medyków Katowice, Poland; Department of Ecology and Biogeography, Faculty of Biological and Veterinary Sciences, Nicolaus Copernicus University in Toruń, Lwowska Toruń, Poland; Department of Ecology and Biogeography, Faculty of Biological and Veterinary Sciences, Nicolaus Copernicus University in Toruń, Lwowska Toruń, Poland; Department of Ecology and Biogeography, Faculty of Biological and Veterinary Sciences, Nicolaus Copernicus University in Toruń, Lwowska Toruń, Poland; Department of Ecology, Faculty of Environmental Sciences, Czech University of Life Sciences Prague, Prague-Suchdol, Czech Republic; Faculty of Science, Charles University in Prague, Prague, Czech Republic; Forensic DNA Service, Prague, Czech Republic; Faculty of Science, Charles University in Prague, Prague, Czech Republic; Forensic DNA Service, Prague, Czech Republic; 2nd Faculty of Medicine. Charles University in Prague, Prague, Czech Republic; Department of Forensic Medicine, Bulovka University Hospital, Prague, Czech Republic; Centre for Advanced Technologies, Adam Mickiewicz University in Poznań, Poznań, Poland; Laboratory of Criminalistics, Adam Mickiewicz University in Poznań, Poznań, Poland

**Keywords:** forensic sciences, forensic entomology, costal cartilage, DNA barcoding, foramen nutrients, tooth cavity, nutrient canal

## Abstract

The study aimed to analyze the entomological material collected during 13 autopsies performed on the unidentified cadavers revealed at different stages of decay in the Upper Silesia Region (Poland) over 2016–2022. During the preparation of human tissues for genetic identification, we revealed larvae, puparia, and adult insects in previously undescribed locations: costal cartilage, femur nutrient canals (*foramen nutrients*), and tooth cavities. The taxonomical assessment was done using morphological examination or DNA barcoding, where necessary. Based on our observations, we conclude that the apical constriction, foramen, and cavities may serve as migration paths inside teeth, and the femur nutrient canals to the bone marrow. The study also revealed that the beetle *Necrobia ruficollis* (Fabricius, 1775) and the moth family Pyralidae Latreille, 1802 (Phycitinae) moths can form pupal chambers inside the costal cartilage, indicating that these insects can complete their life cycle inside this cache. We believe that the newly reported locations of carrion insects in human remains may be relevant to forensic entomology, as they provide new opportunities to collect insect evidence.

**Key points:**

- Costal cartilage may serve as an occasional cache for adults and immatures of carrion insects.
- Tooth cavities and apical foramen may serve as entryways for necrophilous insect larvae.
- Insect larvae use nutrient canals as migratory pathways to the bone marrow.

## Introduction

Entomological material, is often collected directly at the crime scene, following established protocols [1, 2]; however, in some cases, entomological material may not be visible during a typical cadaver examination, and only specialist preparation, such as cleaning tissue and collecting material for human DNA isolation, may reveal the potential presence of insect evidence [3]. Costal cartilages, teeth, and femurs are the most common sampled tissues from highly decomposed human cadavers. Cartilage tissues may serve as an alternative forensic material, especially for forensic genetic purposes, like DNA typing [4–6] and epigenetic age prediction [7, 8]. DNA isolated from costal cartilages is not degraded, which makes it possible to obtain a complete genetic profile even from highly decomposed or almost completely skeletonized cadavers. Since the costal cartilage is one of the last remaining tissues preceding complete skeletonization [6, 9], cartilage tissues are increasingly used in forensic sciences [6, 10–13].

However, in the case of complete skeletonization, when the costal cartilage is no longer available, fragments of the femur or teeth are sampled for genetic testing [14]. The initial step in preparing the material is the removal of any decomposed soft tissues, if their remains are still present. For costal cartilages, decomposed muscles must be removed, while for femur fragments, both decomposed muscles and bone marrow must be cleaned [15–17]. While removing the surface layer, the presence of entomological material on the surface of the cartilage and in its core, i.e. nutritional channels, can be revealed. In order to obtain smaller fragments of bones and teeth, the material is frequently split into pieces with a hammer or a circular saw. In the case of teeth, their fragmentation is essential for exposing the pulp cells that are an important source of DNA [18]. While there is no need to destroy the tooth crown to obtain DNA, as it can be avoided by taking an X-ray image [19]. At this stage of the teeth preparation, insect larvae may be revealed in the medullary cavity [3]. Examination of these niches for necrophagous insects may reveal hidden entomological evidence that may provide a more accurate estimation of the post-mortem interval (PMI), particularly for highly decomposed or skeletonized cadavers [20].

The current study aimed to examine entomological evidence revealed in the material sampled post-mortem from unidentified human bodies in hitherto undescribed locations: costal cartilages, teeth, and femur nutrient canals (*foramen nutrients*). Insect samples were collected during medico-legal autopsies ordered by the Prosecutor's Office in the course of investigations aimed at personal identification.

## Material and methods

### Study material and sample collection

The study material originated from the unidentified bodies revealed in the Upper Silesia Region (Poland) over 2016–2022, which were subjected to medico-legal autopsies commissioned by the Prosecutor’s Office. The samples were collected with the Prosecutor’s Office and the Local Bioethical Commission consents. In sum, 12 costal cartilage fragments, 3 teeth, and 1 femur fragment were collected from 13 cadavers (11 males and 2 females, aged 28–90 years old) at different stages of decay (advanced/skeletonized). The cadaver’s stage of decay was evaluated by a forensic pathologist based on morphological characteristics described by Megyesi et al. [21]. Costal cartilage fragments of approximately 5 cm × 6 cm were sampled from the rib arches. All samples were stored at −20°C until genetic identification analysis was possible. The entomological material revealed during the preparation of the autopsy material was preserved in 70% ethanol.

### Entomological analysis

Insect evidence was identified using the author’s collections and identification keys [22–28]. Voucher specimens have been deposited in the institutional collection of the Department of Ecology and Biogeography, Nicolaus Copernicus University in Toruń, and Laboratory of Criminalistics, Adam Mickiewicz University (UAM) in Poznań.

DNA barcoding was applied for insect species identification when taxonomic assessment was unsuccessful due to material conditions or a lack of discriminatory characteristics. DNA barcoding was carried out according to the protocol described by Grzywacz et al. [29]. Particularly, genomic DNA was isolated using a DNeasy Blood & Tissue Kit (Qiagen, Valencia, CA, USA) following the manufacturer’s protocol. Isolated DNA was quantified with a Qubit 3.0 fluorometer (Thermo Fisher Scientific, Waltham, MA, USA) using dsDNA High Sensitivity Assay Kit (Life Technologies, Inc., Carlsbad, CA, USA) following the manufacturer’s instructions. Primers TY-J-1460 and C1-N-2191 have been used to amplify the cytochrome c oxidase I gene barcode region in PCR reaction [30]. The amplicons were visualized using electrophoresis in a 1% agarose gel, stained with GelRed (Biotium, Darmstadt, Germany), and photographed with Uvidoc HD6 gel documentation system (UVItec Ltd, Cambridge, UK). The successfully amplified products were purified with AMPure XP (1x beads-to-sample volume ratio) (Beckman Coulter, Carlsbad, CA, USA), re-suspended in TE buffer, and measured for the yielded DNA using a Qubit 3.0 fluorometer. PCR sequencing reactions were carried out using the PCR product of the initial PCR reaction (5–20 ng/μL of template DNA) and the BrilliantDye Terminator v.3.1 Cycle Sequencing Kit (NimaGen B.V., Nijmegen, the Netherlands) according to the manufacturer protocol using identical primer set to the initial PCR (5 mmol/L each). The PCR products were purified by the addition of 0.1 volume of 3 mol/L NaAc, 0.1 volume of 125 mmol/L EDTA and 2.5 volume of 95% ethanol and then resuspended in water. Final products of bidirectional Sanger sequencing were resolved using an automated DNA sequencer at the Laboratory of Molecular Biology Techniques, UAM (Poznań, Poland). Generated sequences were edited and then assembled using SeqMan II ver. 4.0 (DNASTAR; Lasergene, Madison, WI, USA).

Resulting sequences were identified by comparison to sequences available in the National Center for Biotechnology Information database (Bethesda, MD, USA) using the Basic Local Alignment Search Tool and Barcode of Life Data System v4 (BOLD) [29–31]. All newly obtained sequences have been deposited in GenBank (https://www.ncbi.nlm.nih.gov/genbank/) (accession numbers: OR600276–OR600281).

## Results

**Figure 1:**
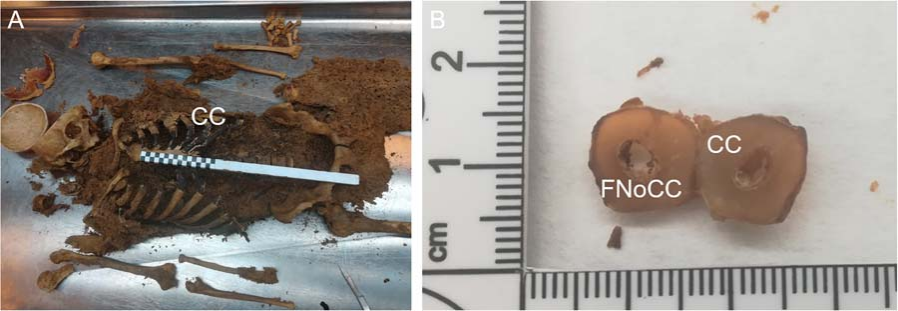
Costal cartilage location on a skeletonized human cadaver with *Dermestes* sp. Larvae, and their exudates and frass of dermestid beetles visible in the thoracic cavity (A) and a section of costal cartilage with the nutrient canal (B). CC: costal cartilage; FNoCC: foramen nutrients of costal cartilage.

During the preparation of autopsy material, the total of 37 samples of entomological material were collected and analyzed. The majority of them (28 out of 37) were related to the costal cartilage: 14 samples were collected from its surface, 12 samples from the costal cartilage nutrient foramen (Figure 1), and 2 samples from between the fascia and the perichondrium.

Six entomological samples were revealed inside the costal cartilage. In two cases, the entomological material was found in the chamber of an intact tooth. In the next two cases, the entomological material adhered to the tooth cavity. In one case, the entomological material was found in the cavity inside the tooth chamber, and in the last case, it was found inside the rib bone. In two cases, insects were recovered from the previously undescribed locations: the inside of the rib bone and the lumen of the femoral nutrient canal.

**Figure 2:**
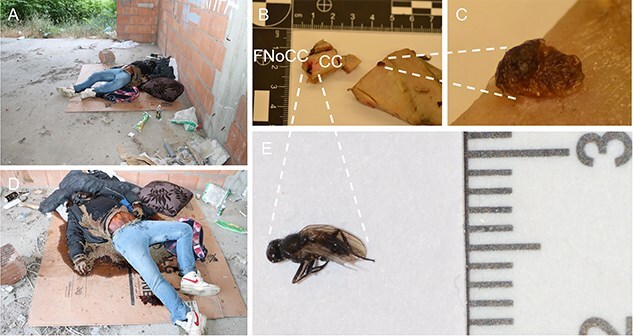
Entomological material collected during the autopsy of an unidentified cadaver revealed in a vacant lot (A, D) in the Upper Silesia Region (Poland) in June 2021: *Fannia canicularis* adult female (E) revealed in the nutrient canal lumen (B) together with the puparium that slid to the surface of the costal cartilage during preparation (C). CC: costal cartilage; FNoCC: foramen nutrients of costal cartilage.

**Figure 3:**
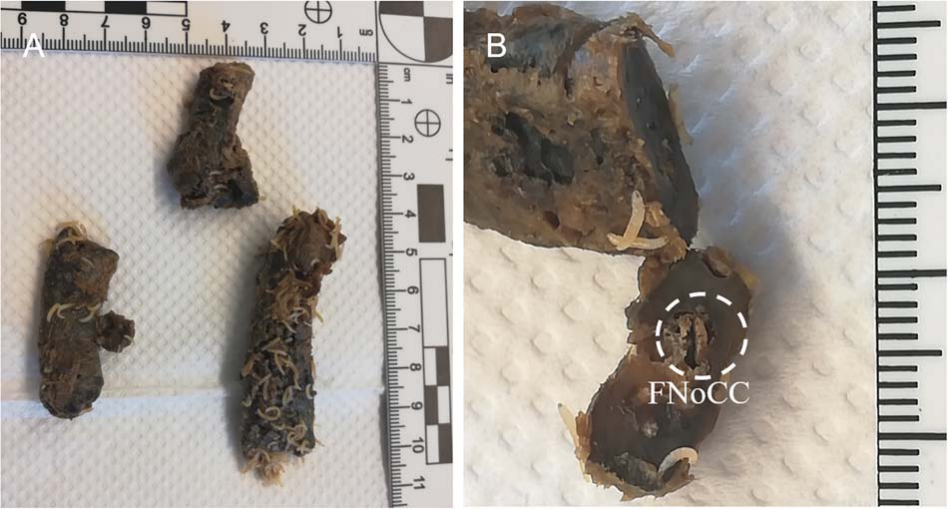
*Stearibia nigriceps* larvae on the costal cartilage surface (LII, *n* = 25) (A) and in the costal cartilage nutrient canal (LIII, *n* = 1) (B) revealed during the autopsy of an unidentified cadaver revealed in a forest in the Upper Silesia Region (Poland) in August 2021. FNoCC: foramen nutrients of costal cartilage; LII: the second instar larvae; LIII: the third instar larvae.

**Figure 4:**
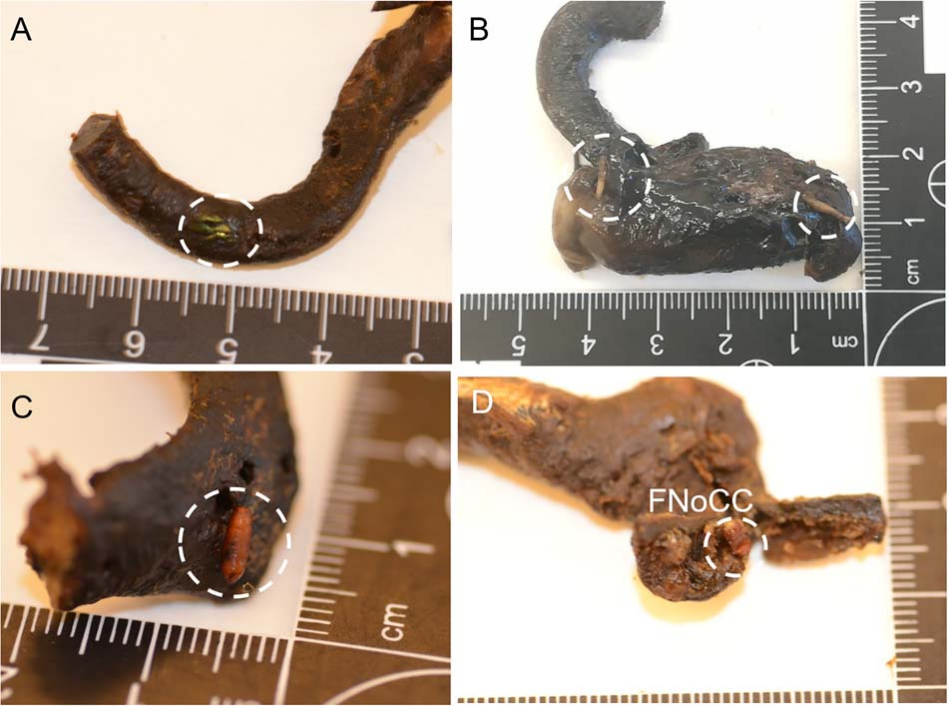
Entomological material revealed during the autopsy of an unidentified cadaver revealed in an apartment in the Upper Silesia Region (Poland) in October 2020. (A) The abdomen of *Chrysomya albiceps* male on the costal cartilage surface. (B) Larvae of *Hydrotaea capensis* (LIII, *n* = 2) on the costal cartilage surface. (C) The puparium of *H. capensis* revealed on the outer surface of the costal cartilage. (D) The puparium of *H. capensis* revealed in the nutrient canal after dissection. FNoCC: foramen nutrients of costal cartilage, LIII: the third instar larvae.

**Figure 5:**
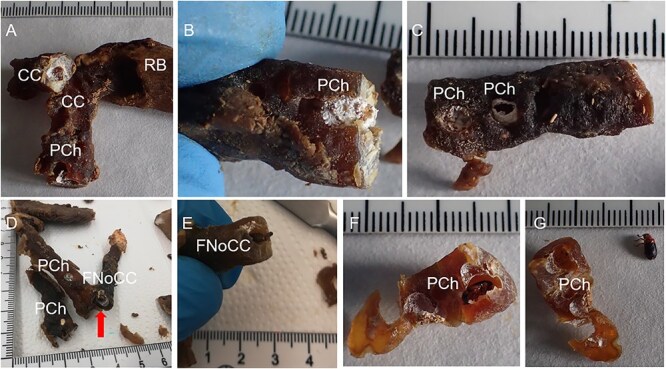
Different developmental stages of *Necrobia ruficollis* revealed in the costal cartilage during the autopsy of an unidentified cadaver revealed in an apartment in the Upper Silesia Region (Poland) in January 2021. (A) Fragment of the costal cartilage protruding from the bony part of the rib with visible pupal chambers. (B) Single pupal chamber with visible superficial white sealing. (C) Fragment of costal cartilage with two pupal chambers. (D) Cross-section of costal cartilage with a visible nutrient canal with revealed pupa (marked with red arrow). (E) Larva revealed in the lumen of the costal cartilage nutrient canal. (F, G) Imago revealed in the lumen of the costal cartilage nutrient canal probably adapted as a pupal chamber entrapping the adult insect. CC: costal cartilage; PCh: pupal chamber; FNoCC: foramen nutrients of costal cartilage; RB: rib bone.

**Figure 6:**
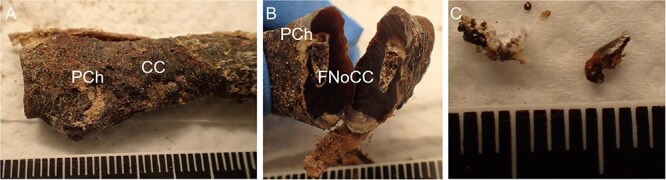
*Necrobia ruficollis* revealed in the costal cartilage of an unidentified cadaver revealed in a basement in the Upper Silesia Region (Poland) in November 2022. (A) The visible outer part of the pupal chamber and the visible surface white sealing of the costal cartilage fragment. (B) Pupal chamber inside. (C) *Necrobia ruficollis* imago (*n* = 2) dissected from the pupal chamber that entrapped the adult insects. CC: costal cartilage; FNoCC: foramen nutrients of costal cartilage; PCh: pupal chamber.

**Figure 7:**
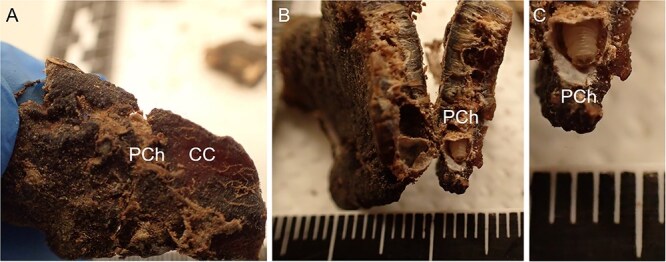
The process of revealing Pyralidae (Phycitinae) larvae in the costal cartilage of an unidentified cadaver revealed in a basement in the Upper Silesia Region (Poland) in November 2022. (A) The visible outer part of the pupal chamber and the visible surface yellowish sealing of the costal cartilage fragment. (B) The inside of the pupal chamber. (C) Close-up of a Pyralidae (Phycitinae) larva in the pupal chamber. CC: costal cartilage; PCh: pupal chamber.

**Figure 8:**
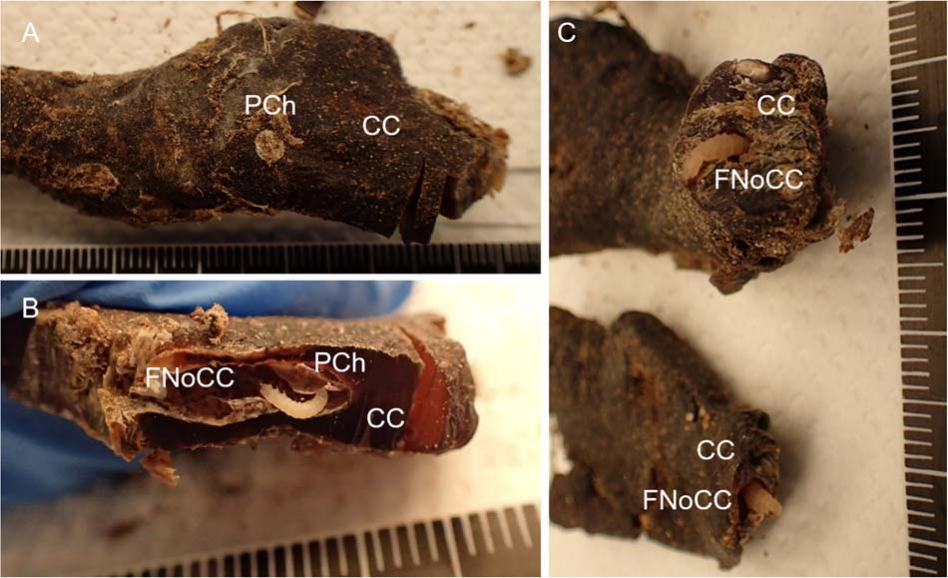
Larvae of the Pyralidae (Phycitinae) family revealed in the pupal chamber (A and B) and in the nutritional canal (C) of the costal cartilage of an unidentified cadaver revealed in a basement in the Upper Silesia Region (Poland) in November 2021. (A) The visible outer part of the pupal chamber and the visible surface white sealing of the costal cartilage fragment. (B) The pupal chamber inside with Pyralidae (Phycitinae) larva. (C) Pyralidae (Phycitinae) larvae in the costal cartilage nutrient canal. CC: costal cartilage; FNoCC: foramen nutrients of costal cartilage; PCh: pupal chamber.

The procedure of human tissue preparation for genetic identification revealed larvae, puparia, and adult insects in previously undescribed locations. These were blowfly larvae of *Calliphora vomitoria* (Linnaeus, 1758), *Lucilia caesar* (Linnaeus, 1758), *Lucilia sericata* (Meigen, 1826), *Phormia regina* (Meigen, 1826), and *Protophormia terraenovae* (Robineau-Desvoidy, 1830), larvae and puparium of a muscid fly *Hydrotaea capensis* (Wiedemann, 1818), larvae of skipper flies *Stearibia nigriceps* (Meigen, 1826), and Hymenoptera pupae, which were found on the costal cartilage surface. Inside the cartilage’s nutrient canal, an adult female of a fanniid fly *Fannia canicularis* (Linnaeus, 1761) (Figure 2E)*,* a larva of a skipper fly *S. nigriceps* (Figure 3B), puparium of a muscid fly *H. capensis* (Figure 4D), larvae and imago of a ham beetle *Necrobia ruficollis* (Fabricius, 1775) (Figures 5E, 5F, 6B, and 6C), and larvae of Pyralidae moths (Phycitinae) were revealed (Figures 7 and 8).

The white spots observed on the surface of the cartilage indicated the presence of a pupal chamber, and using a scalpel helped to prepare the pupal chamber and reveal the enclosed entomological material (Figures 5B, 6A, 7A, 8A). White caps were probably the entrance to the pupal chambers.

**Figure 9:**
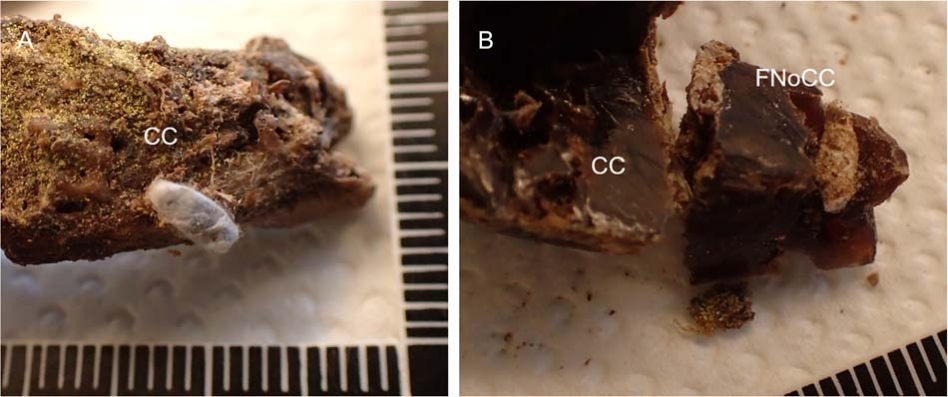
Braconidae, *Aspilota* sp. pupae in cocoons revealed on the costal cartilage surface (A) and in the nutritional canal (B) of the costal cartilage of an unidentified cadaver revealed in a basement in the Upper Silesia Region (Poland) in November 2021. CC: costal cartilage; FNoCC: foramen nutrients of costal cartilage.

Material belonging to Braconidae, *Aspilota* sp. Förster, 1863 was revealed both on the costal cartilage surface and inside cartilage (Figure 9A and B).

**Figure 10:**
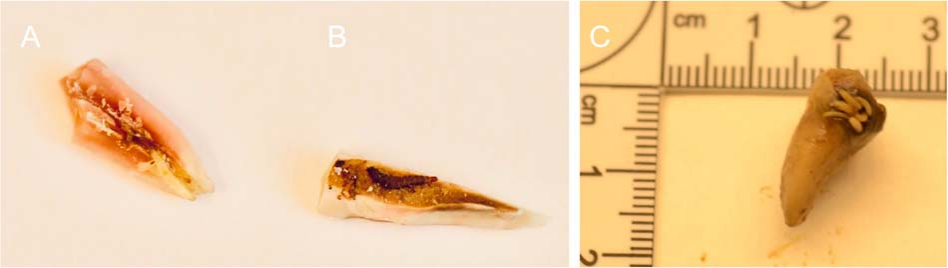
Larvae revealed during the teeth preparation of unidentified cadavers revealed in forests in the Upper Silesia Region (Poland) in October 2016 (A, B) and October 2021 (C). (A) Fly larva, most likely of *Stearibia* sp., Piophilidae. (B) *Necrobia* beetle larva of the Cleridae family inside the tooth cavity. (C) *Stearibia nigriceps* adjacent to the cavity (LIII larvae, *n* = 7) and inside of the tooth crown (LIII larvae, *n* = 5, not visible). LIII: the third instar larvae. The Piophilidae larvae were revealed in the femur’s foramen nutrients, which suggests how the larvae might migrate to the bone marrow (Figure 11).

Larvae of the genus *Necrobia* Olivier, 1795 and the family Piophilidae were revealed in the tooth cavity and a healthy tooth, respectively (Figure 10A and B). The migration path to the tooth chamber most likely led through the apical foramen and apical constriction. Our observations have also shown that the larvae of *S. nigriceps* can penetrate the tooth through a cavity in the tooth crown (Figure 10C).

**Figure 11:**
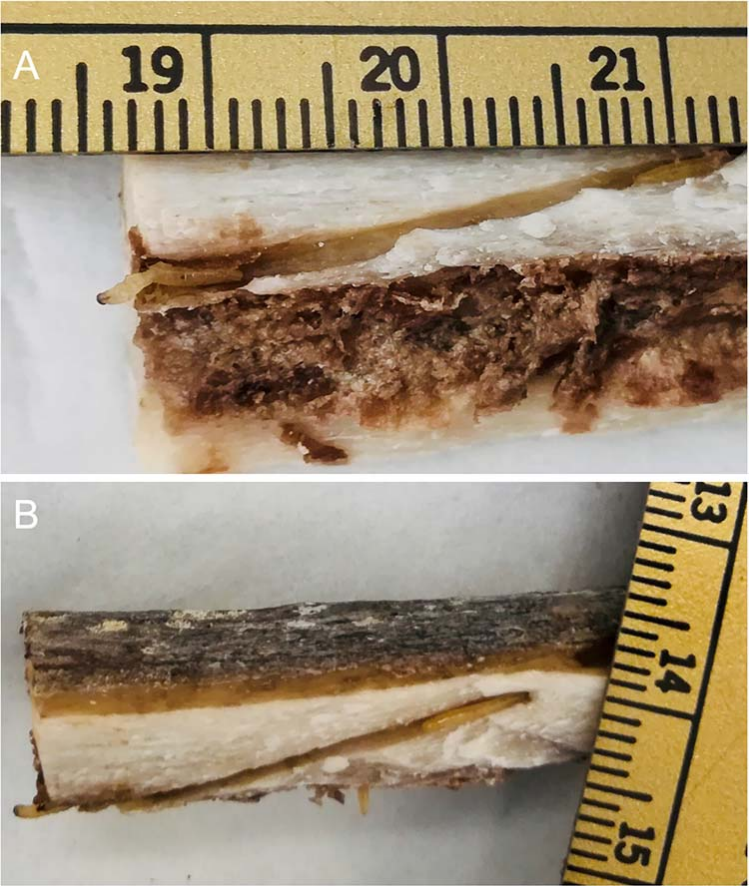
Piophilidae larvae revealed in the nutrient canal of the femoral shaft of an unidentified cadaver revealed in the bushes in the Upper Silesia Region (Poland) in March 2018. (A) Larva migrating to the medullary cavity, visible at the edge of the nutrient canal-to-medullary cavity opening. (B) Larva migrating from the medullary cavity to the feeding hole entrance in the femur.

During the preparation, we also discovered an incomplete entomological material, e.g. the abdomen of blowfly *Chrysomya albiceps* (Wiedemann, 1819) male (Figure 4A), and remains of the anterior body part with a cephaloskeleton of *S. nigriceps* LII larvae. In some cases, DNA barcoding was performed to confirm the species identification. Details of the entire entomological material revealed from the individual cadavers included in the presented study are presented in Table 1.

**Table 1:** Details of the entomological material collected from the individual, unidentified cadavers at different stages of decay revealed in the Upper Silesia Region (Poland) over 2016–2022.

Cadaver details						Case details			
No	Order	Family	Species^*^	Developmental stage	Location on the body	Date of discovery	Sex (F or M)/age (years)	Decomposition stage	Discovery place
1	Coleoptera	Cleridae	N/A	Larva	Inside undamaged tooth	Oct, 2016	F/28	Advanced decomposition	Forest, under the tree
2	Coleoptera	Cleridae	*Necrobia ruficollis*	Larva	Inside costal cartilage	Oct, 2019	M/40	Advanced decomposition	Vacant building
3	Coleoptera	Cleridae	*Necrobia ruficollis*	Larva	Costal cartilage surface	Oct, 2019	M/40	Advanced decomposition	Vacant building
4	Coleoptera	Cleridae	*Necrobia ruficollis*	Imago	Inside costal cartilage	Jan, 2021	M/UD	Skeletonized	Apartment
5	Coleoptera	Cleridae	*Necrobia ruficollis*	Larva	Costal cartilage surface	Jan, 2021	M/UD	Skeletonized	Apartment
6	Coleoptera	Cleridae	*Necrobia ruficollis*	Larva	Inside costal cartilage	Jan, 2021	M/UD	Skeletonized	Apartment
7	Coleoptera	Cleridae	*Necrobia ruficollis*	Larva	Inside of the rib bone	Jan, 2021	M/UD	Skeletonized	Apartment
8	Coleoptera	Cleridae	*Necrobia ruficollis*	Larva	Inside costal cartilage	Jul, 2022	M/59	Skeletonized	Shelter in the forest
9	Coleoptera	Cleridae	*Necrobia* sp.	Puparium	Inside costal cartilage	Jul, 2022	M/59	Skeletonized	Shelter in the forest
10	Coleoptera	Cleridae	*Necrobia ruficollis*	Imago	Inside costal cartilage—pupal chamber	Nov, 2022	M/70	Skeletonized	Basement
11	Coleoptera	Dermestidae	N/A	Larva	Costal cartilage surface	Oct, 2019	M/40	Advanced decomposition	Vacant building
12	Diptera	Calliphoridae	*Calliphora vomitoria*	Larva LIII	Surface between the perichondrium and the fascia	Feb, 2022	M/29	Advanced decomposition	Forest
13	Diptera	Calliphoridae	*Calliphora vomitoria*	Larva LIII	Surface between the perichondrium and the fascia	Dec, 2020	M/41	Advanced decomposition	Bushes near the river bed
14	Diptera	Calliphoridae	*Chrysomya albiceps*	Abdomen of male imago	Costal cartilage surface	Oct, 2020	F/90	Advanced decomposition	Apartment
15	Diptera	Calliphoridae	*Lucilia caesar* ^*^	Larva	Costal cartilage surface	Jun, 2021	M/38	Advanced decomposition	Vacant building in the city center in wastelands proximity
16	Diptera	Calliphoridae	*Lucilia sericata* ^*^	Larva	Costal cartilage surface	Jun, 2021	M/38	Advanced decomposition	Vacant building in the city center in wastelands proximity
17	Diptera	Calliphoridae	*Phormia regina* ^*^	Larva	Costal cartilage surface	Jun, 2021	M/38	Advanced decomposition	Vacant building in the city center in wastelands proximity
18	Diptera	Calliphoridae	*Protophormia terraenovae* ^*^	Larva	Costal cartilage surface	Jun, 2021	M/38	Advanced decomposition	Vacant building in the city center in wastelands proximity
									
19	Diptera	Calliphoridae	*Protophormia terraenovae*	Puparium	Tooth’s surface	Sep, 2022	M/48	Advanced decomposition	Bushes
20	Diptera	Fanniidae	*Fannia canicularis* ^*^	Imago	Inside costal cartilage	Jun, 2021	M/38	Advanced decomposition	Vacant building in the city center in wastelands proximity
21	Diptera	Muscidae	*Hydrotaea capensis*	Larva LIII	Costal cartilage surface	Oct, 2020	F/90	Advanced decomposition	Apartment
22	Diptera	Muscidae	*Hydrotaea capensis*	Puparium	Inside costal cartilage	Oct, 2020	F/90	Advanced decomposition	Apartment
23	Diptera	Muscidae	*Hydrotaea capensis*	Puparium	Costal cartilage surface	Oct, 2020	F/90	Advanced decomposition	Apartment
24	Diptera	Piophilidae	N/A	Larva	Inside undamaged tooth	Oct, 2016	F/28	Advanced decomposition	Forest, under the tree
25	Diptera	Piophilidae	N/A	Larva	Femur, inside foramen nutrients	March, 2018	M/30	Skeletonized	Bushes
26	Diptera	Piophilidae	*Stearibia nigriceps*	Larva LIII	Costal cartilage surface	Oct, 2019	M/40	Advanced decomposition	Vacant building
27	Diptera	Piophilidae	*Stearibia nigriceps*	Larva	Costal cartilage surface	Aug, 2021	M/60	Sdvanced decomposition	Forest
28	Diptera	Piophilidae	*Stearibia nigriceps*	Larva	Inside costal cartilage	Aug, 2021	M/60	Advanced decomposition	Forest
29	Diptera	Piophilidae	*Stearibia nigriceps*	Larva LIII	Costal cartilage surface	Oct, 2021	M/30	Advanced decomposition	Forest
30	Diptera	Piophilidae	*Stearibia nigriceps*	Larva LIII	Inside costal cartilage	Oct, 2021	M/30	Advanced decomposition	Forest
31	Diptera	Piophilidae	*Stearibia nigriceps*	Larva LIII	Adhered to the tooth cavity	Oct, 2021	M/30	Advanced decomposition	Forest
32	Diptera	Piophilidae	*Stearibia nigriceps*	Larva LIII	Inside the tooth crown	Oct, 2021	M/30	Advanced decomposition	Forest
33	Diptera	Piophilidae	*Stearibia nigriceps*	Remnant of LII larva	Adhered to the tooth cavity	Oct, 2021	M/30	Advanced decomposition	Forest
34	Hymenoptera	Braconidae	*Aspilota* sp.	Pupa in cocoon	Inside costal cartilage	Nov, 2022	M/70	Skeletonized	Basement
35	Hymenoptera	Braconidae	*Aspilota* sp.	Pupa in cocoon	Costal cartilage surface	Nov, 2022	M/70	Skeletonized	Basement
36	Lepidoptera	Pyralidae (Phycitinae)	N/A	Larva	inside costal cartilage—pupal chamber	Nov, 2022	M/70	Skeletonized	Basement
37	Lepidoptera	Pyralidae (Phycitinae)	N/A	Mature larva	Inside costal cartilage—pupal chamber	Nov, 2022	M/70	Skeletonized	Basement

^*^Identified using DNA barcoding. LII: the second instar larva; LIII: the third instar larva; N/A: not applicable; UD: undetermined. F: female; M: male.

## Discussion

The study presents the results of research on the entomological material collected during autopsies of unidentified human remains discovered in the Upper Silesia Region (Poland) over 6 years (2016–2022). The forensic material sampled for the DNA genotyping comprised mostly fragments of costal cartilage. Costal cartilage is successfully used for forensic genetic identification purposes because its DNA is relatively well protected against degrading environmental factors [4, 6, 32] and additionally free from the chimerism that may occur after bone marrow transplantation [33].

Dermestidae beetles are frequently reported from indoor cadavers in large agglomerations of Central Europe [34]. Current cases also confirmed their presence. The autopsy revealed evidence of the dermestid beetles feeding on the visible soft remnants of costal cartilage, which, apparently, was the last remaining type of non-skeletonized tissue. Numerous studies reported that when the costal cartilage is no longer available on a cadaver, only the hard tissues (i.e. fragments of the femoral shaft, or teeth) can be sampled [35].

We identified a diverse entomofauna in the sampled material. To the best of our knowledge, no previous study has reported on insect evidence found in costal cartilage, femur’s nutrient canals (*foramen nutrients*), or tooth cavities. However, insect evidence was frequently reported from bones in forensic and entomoarcheological studies [3, 27, 36]. Our findings suggest that forensic genetics should examine the inside of costal cartilage, teeth, or bones during a forensic autopsy.

The vast majority of species revealed in the cases analyzed in this study are common necrophagous species such as *L. caesar*, *Linothele sericata*, *P. regina*, *P. terraenovae*, *F. canicularis* [25, 28, 37], or *N. ruficollis* [38]. However, discovering the larval stages of *H. capensis* on the costal cartilage surface and their puparia inside and on the cartilage surface was a relatively interesting finding, as *H. capensis* is not frequently reported from real cases or forensic entomology oriented succession experiments [36, 37]. A larva of *N. ruficollis* bit into the cartilage and, at the right age, produced a hardening secretion that formed the inner walls of the pupal chamber, where it underwent pupation and a subsequent imago eclosion. The imago of *N. ruficollis* revealed inside the pupal chamber in the cartilage shows that the whole pupal development can take place inside the cartilage. It has previously been only described that *N. ruficollis* have the ability to form pupal chambers in cotton balls [38]. Zanetti et al. [39] showed that *Necrobia rufipes* (Fabricius, 1781) can produce taphonomic marks like scratches, pits, holes, and tunnels in integumental, connective, and muscular tissues. Recently, it has been shown that the larder beetle *Dermestes maculatus* De Geer, 1774 larvae may dig pupation chambers in dry human bones dated to medieval times [40]. Our study also showed that *Necrobia* beetles and moths of the Pyralidae (Phycitinae) family are able to create pupal chambers inside the cartilage, so perhaps this ability is more common among necrophilous insects.

In addition to the typical necrophagous species described above, traces of feeding by Braconidae parasitoids were also observed. It is a large taxon, and probably the individuals of the *Aspilota* sp., which were observed on the surface and inside the cartilage, parasitized necrophagous species on the corpse. On this particular corpse, only late colonizing ham beetles and pyralid moths were found, but it is highly probable that other insects were feeding on them earlier. In the aspect of forensic entomology, parasitoids are mainly used to estimate minimum PMI in long PMI cases. So far, most reports used parasitoid wasps of *Nasonia vitripennis* (Walker, 1836) [41, 42]. Braconidae were very infrequently reported from human or pig cadavers [43]. Another phenomenon that we encountered during this study was the penetration of the larvae into the costal cartilage, tooth, or femur with an intact structure. The costal cartilage is the last tissue that remains intact before complete skeletonization of the cadaver. Its nutrient canal diameter can be as large as 0.3 cm, which allows insects to migrate freely and feed on the soft content that fills the canal. Our research showed that one of the locations occupied by insects may be the costal cartilage nutrient canals. They can be a food source, a migration path or a place for the further development during the pupal stage. The diameter of the nutrient canal after cleaning is 3.0–3.5 mm, which allows the larvae of most carrion insects to freely migrate through the canal. As for the intact tooth penetration, since the diameter of the narrowest point of the tooth ranges from 0.24 to 0.47 mm [44], it seems that only the smallest insects (e.g. the first instar larvae) would be able to access the tooth chamber. The average width of a head can be 0.19 mm (range between 0.15 and 0.26 mm) for the first instar larvae of *N. rufipes* [45] and no more than 0.34 mm (own measurements) for the first instar larvae of piophilid flies. Our measurements showed that the diameter of the femur nutrient canal ranged from 0.5 to 2.0 mm. Other studies report that in 65.20% of the measured cases, it was 2 mm; in 22.92% cases, it was 0.5–2.0 mm; and in 11.88% cases, it was up to 0.5 mm [46]. Therefore, natural openings in teeth and canals in bones allow necrophagous insects to inhabit the tooth chambers or medullary cavities.

Finally, our findings highlight the importance of paying attention to the fragments or remains of classical entomological evidence when examining human remains. Insect fragments can support the forensic investigation, especially in the cases of cadavers at a highly advanced decomposition stage. The paper emphasizes the need for collaboration between specialists representing various fields of forensic sciences and describes new locations in human remains that may be relevant for forensic entomology. Forensic geneticists preparators may find valuable entomological evidence during the costal cartilage, teeth, and bones preparation for the DNA isolation. Another possible application of forensic entomology is in the emerging field of wildlife forensics, where the entomological evidence can link the animal remains (bush meat, tanned hide, bones, teeth, claws, etc.) to the geographic area [47, 48]. The entomological material should thus always be collected for further forensic entomological analysis.

The presented study is not devoid of limitations. Most of the analyzed material came from unidentified corpses (often homeless people) in an extremely advanced stage of decomposition. Material delivered for DNA analyses consisted only of cartilage or bone/teeth sections. These two facts combined with the limited information from the Prosecutor's Office prevented the PMI estimation in the analyzed cases, which is the main limitation of this study.

## Conclusions

Costal cartilage may constitute a cache for insect preimaginal stages. The tooth cavity and apical foramen may serve as a colonization gate for larvae of necrophilous insects. The femur nutrient canal (foramen nutriens) constitutes a migratory pathway to the bone marrow. A collaboration between forensic geneticists and entomologists may help to reveal new insect niches, which might be beneficial for the practice and research of forensic entomology.
